# Seeded Growth Synthesis of Zirconia@Gold Particles in Aqueous Solution

**DOI:** 10.3390/nano10061197

**Published:** 2020-06-19

**Authors:** Gregor Thomas Dahl, Jan-Dominik Krueger, Sebastian Döring, Horst Weller, Tobias Vossmeyer

**Affiliations:** 1 Institute of Physical Chemistry, University of Hamburg, Grindelallee 117, 20146 Hamburg, Germany; dahl@chemie.uni-hamburg.de (G.T.D.); jadohelm@gmail.com (J.-D.K.); sebastiandoering@t-online.de (S.D.); horst.weller@chemie.uni-hamburg.de (H.W.); 2 Fraunhofer Center for Applied Nanotechnology CAN, Grindelallee 117, 20146 Hamburg, Germany

**Keywords:** zirconia mesoparticles, gold nanoparticles, metal-ceramic composite, zirconia@gold, seeded growth, gold nanoshell, nanoparticle immobilization, aminomethylphosphonic acid

## Abstract

Metal-ceramic composite particles are of increasing interest due to their potential applications in photonic metamaterials as well as next-generation catalysts. The zirconia-gold system has received little attention due to the lack of controllable preparation methods. Well-known methods for the deposition of gold nanoshells on silica spheres, however, should be adaptable for similar zirconia-based materials. Here, we present a novel synthetic approach to the well-controlled deposition of gold on the surface of sol-gel derived zirconia mesoparticles by a stepwise method involving the immobilization of gold nanoparticles and repeated seeded-growth steps. We show that the immobilization efficiency is strongly enhanced by acidification with hydrochloric acid and additional employment of aminomethylphosphonic acid as coupling agent. The optimum conditions are identified and the subsequent incremental growth by seeded reduction of gold is demonstrated. The results shed light on the parameters governing the preparation of zirconia@gold composite particles and our synthetic approach provides a promising tool for future developments in complex nanomaterials design.

## 1. Introduction

Metal-ceramic composite materials have attracted significant interest among different scientific communities during the past years. When designed with precise geometries, they show exceptional tunable optical properties as reported, e.g., for gold nanoshells and patchy particles [1,2]. As a consequence, their potential use in advanced functional materials is discussed in view of various applications, e.g., biosensing, surface-enhanced spectroscopy, and thermophotovoltaics [3,4]. At the same time, such composite nanomaterials seem particularly appealing to researchers focusing on heterogeneous catalysis. Here, the ceramic component often acts as support material as it can be prepared with high specific surface area and since it provides stabilization of the active nanoparticular metal by permanent immobilization [5]. Among others, the combination of zirconia with gold has raised interest for its catalytic potential, e.g., in the water-gas shift reaction [6]. Zirconia as a support material is particularly promising due to its high thermal stability and chemical inertness [7,8]. However, an involvement of the ceramic support in catalytic mechanisms and enhanced catalytic performance of active sites at the metal-ceramic interface are also a matter of discussion [9]. While most materials in this context are prepared by simple techniques, e.g., co-precipitation [6,10,11] and deposition precipitation [12,13,14], the immobilization of colloidal gold has only been addressed incidentally [15]. All reported methods allow only little or no control over the morphology or the precise dimensions of the material features. Moreover, the deposition of gold on spherical zirconia meso- and microparticles has not been dealt with, although gold deposition on silica nanospheres has been intensely studied for many years [1,16,17,18,19,20]. Monodisperse spherical zirconia particles have become available recently by substantially improved sol-gel routes [21,22,23,24], providing a new kind of base materials for the preparation of innovative metal-ceramic nanomaterials. However, the controlled growth of metal nanoshells on oxide particles is known to pose a major challenge and requires the individual design and fine-tuning of the preparation process for each combination of materials.

**Figure 1:**
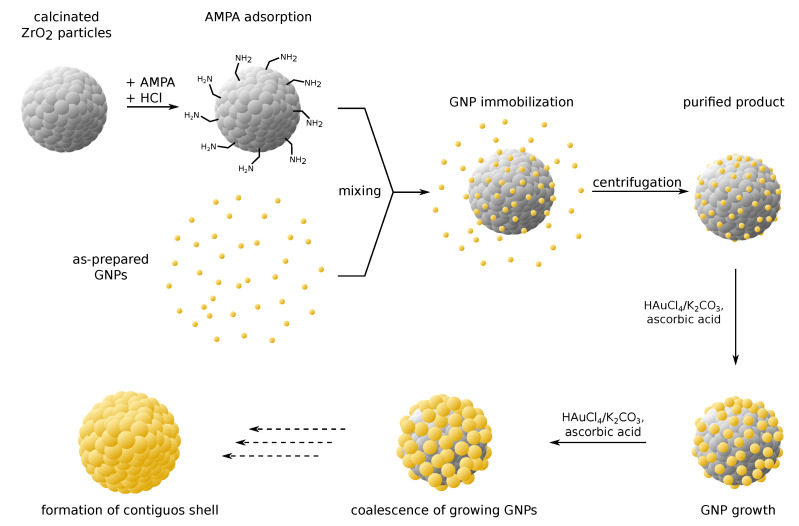
Synthesis strategy for zirconia@gold composite particles. The preparation follows a multi-step route of gold nanoparticle (GNP) immobilization followed by repeated seeded growth. Aminomethylphosphonic acid (AMPA) and acidification with hydrochloric acid enable effective coupling of GNPs with the zirconia surface. Successive GNP growth results in the formation of a gold nanoshell.

In this communication, we report the successful deposition of homogeneously dispersed gold nanoparticles (GNPs) on zirconia mesoparticles by an ecofriendly aqueous method. Here, changes to the pH by addition of hydrochloric acid are shown to greatly impact the deposition efficacy and allow for considerable improvements of the process. The employment of aminomethylphosphonic acid (AMPA) as coupling agent is shown to additionally promote GNP immobilization. Furthermore, we present results on the subsequent deposition of additional gold via seeded growth, using the immobilized GNPs as reduction nuclei. Based on the literature on silica@gold nanoshell synthesis, we assume that this approach should enable the successive growth of surface-attached GNPs and eventually lead to the formation of a contiguous gold nanoshell. The entire multi-step synthesis strategy is illustrated in Figure 1. While the general synthetic concept is based on a protocol for silica@gold composite particles [1], we adapted the method for zirconia particles and introduced a novel coupling approach. To the best of our knowledge, no protocol for the controlled immobilization of GNPs and the seeded growth deposition of gold on monosized spherical zirconia particles has been reported in the literature, to date.

## 2. Results and Discussion

For the preparation of zirconia@gold composite particles, spherical zirconia mesoparticles were used as starting material. They were obtained with an as-prepared mean diameter of 384±36 nm following a protocol that was previously published by our group [24], calcined at 600 °C, and redispersed before further use. This particle size is particularly desirable for facile purification during synthesis or for recovery in potential catalytic applications, while still maintaining a high surface-to-volume ratio. In order to study the effect of acidification and the use of AMPA as coupling agent on the immobilization of GNPs on the surface of zirconia mesoparticles, a systematic series of experiments was conducted, as summarized in Table S1 in the Supplementary Materials. Here, particle samples were mixed with varying amounts of hydrochloric acid, followed by the addition of small GNPs (Ø (2.8±0.9) nm) prepared by the Duff method [25]. For each sample, the pH was determined experimentally. An equivalent series of experiments was conducted with the additional use of AMPA at a fixed concentration. After a reaction time of half an hour the particles were purified by centrifugation, effectively removing excess GNPs in the solution. Hence, subsequently conducted characterizations of the samples by scanning electron microscopy (SEM), atomic absorption spectroscopy (AAS), and optical spectroscopy (UV/vis/NIR) considered only the remaining solid material, i.e., zirconia mesoparticles, immobilized GNPs, and potentially larger GNP aggregates, if present. Additionally, sample concentrations were determined gravimetrically. In the following, the most relevant results of some selected samples are presented and discussed. However, complete characterization results of all samples and tabulated data are provided in the Supplementary Materials.

**Figure 2:**
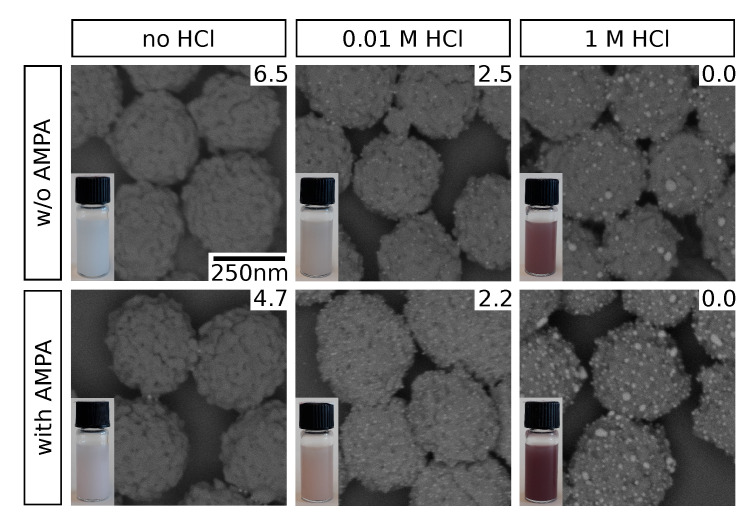
Scanning electron micrographs of GNP-decorated zirconia particles, obtained using varying HCl concentrations during GNP immobilization and absence or presence of AMPA. An energy selective backscatter (ESB) detector was used, yielding a strong contrast between zirconia (dark) and gold (bright). Photographs of the particle dispersions are shown in the insets, pH values were determined experimentally and are indicated accordingly (top right corner). The scale bar shown in the top left image applies to all micrographs.

Representative electron micrographs of selected samples are shown in Figure 2. Clearly, pH adjustment by HCl addition promotes the immobilization of GNPs on the zirconia particle surface. However, at high HCl concentrations (1 M), significant increase in size of the surface-attached GNPs is observed. We attribute this to GNP aggregation as a consequence of the high acidity. These observations based on SEM data are supported by the color impression of the purified particle suspensions (insets). While they appear whitish when no HCl is added, a distinct brownish tinge is observed at 0.01 M HCl, where substantial amounts of GNPs are evidently immobilized. This corresponds to the typical surface plasmon resonance (SPR) absorption for GNPs in this size range [25], giving prove for their permanent immobilization as well as the nonappearance of significant coalescence which would result in a distinct spectral red-shift (see Supplementary Materials, Figure S2) [26]. The latter is, hence, observed for high HCl concentrations: a strong red to purple coloration of the particle suspensions indicates this sort of SPR band shift as a result of GNP aggregation. We attribute the observed effect to changes in the electrostatic interactions. On the one hand, the zirconia surface exhibits a net positive charge at a pH below its isoelectric point of 6.7 to 6.9 [27,28] and electrostatic attraction to the negatively charged GNPs [15] occurs. Similar effects have been observed previously for GNP immobilization on other amine-functionalized surfaces [29,30]. On the other hand, acidification compromises the electrostatic stabilization of the GNPs which additionally facilitates their deposition on the zirconia surface. The latter, however, also promotes GNP aggregation, as observed at higher HCl concentrations in this study. Our findings are in agreement with increased affinities of GNPs and metal complexes to oxide surfaces in acidic solution, as reported in the literature [15,31].

Besides the effect of HCl addition, it is also observed that the application of AMPA as coupling agent additionally improves GNP immobilization, as indicated by SEM data and the coloration of the purified suspensions compared to those obtained in absence of AMPA. While this observation could possibly be explained by the acidity of AMPA at low HCl concentrations (see pH, Figure 2), this effect should be negligible at higher HCl concentrations. Therefore, we assume that AMPA functions as a coupling agent between the zirconia surface and the GNPs. This interpretation is in agreement with known characteristics of the involved functional groups, i.e., the affinity of phosphonic acids to metal oxide surfaces [32,33], and the electrostatic attraction of amines and (negatively charged) GNPs [34,35]. The simultaneous addition of HCl presumably supports this coupling effect by establishing a favorable pH for optimal protonation states of the ampholytic AMPA for electrostatic attraction to both the zirconia surface and the GNPs.

**Figure 3:**
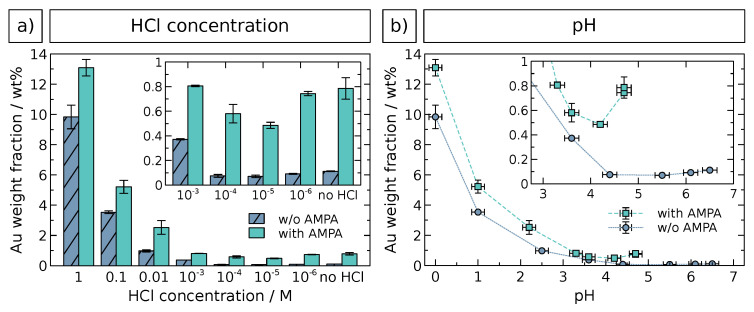
AAS elemental analysis of GNP-decorated zirconia particles, as a function of (**a**) HCl concentration and (**b**) pH. Vertical error bars indicate the standard deviation of multiple measurements or weighing error (whichever is larger), horizontal error bars represent the uncertainty of pH determination. The insets show details of data at lower HCl concentration or higher pH values, respectively.

The extent of GNP immobilization was quantified by AAS elemental characterization of all samples. The results are presented in Figure 3. Gold weight fractions were evaluated as a function of both the HCl concentration (Figure 3a) and the pH value (Figure 3b) as determined experimentally, since the latter is also affected by the other pH-active components, e.g., AMPA and the zirconia surface. The data confirm the trends observed by SEM analysis and underline the more effective immobilization of GNPs at HCl concentrations ≥0.01 M or pH ≤ 2.2 and in presence of AMPA. High gold weight fractions at ≥0.1 M HCl are most likely due to undesirable immobilized GNP aggregates (see Figure 2) and, possibly, also caused by free GNP aggregates in solution, which could not be separated during purification. At lower HCl concentrations and higher pH, very small Au fractions with negligible differences are observed, as shown by the insets. Very similar results were obtained by quantitative UV/vis/NIR analysis (see Figure S2 in the Supplementary Materials). Here, the aggregation hypothesis is substantiated by a significant red shift of the SPR band at high HCl concentrations. An optimal HCl concentration for GNP immobilization using the method presented here was therefore found to be approximately ∼0.01 M, since it is high enough to drastically enhance the immobilization, but still low enough to prevent GNP aggregation. Aggregation is undesired with respect to both the successive controlled growth of gold and the potential employment as a catalyst material. Moreover, the presence of 6 mM AMPA revealed an additional promoting effect for GNP immobilization under these conditions.

**Figure 4:**
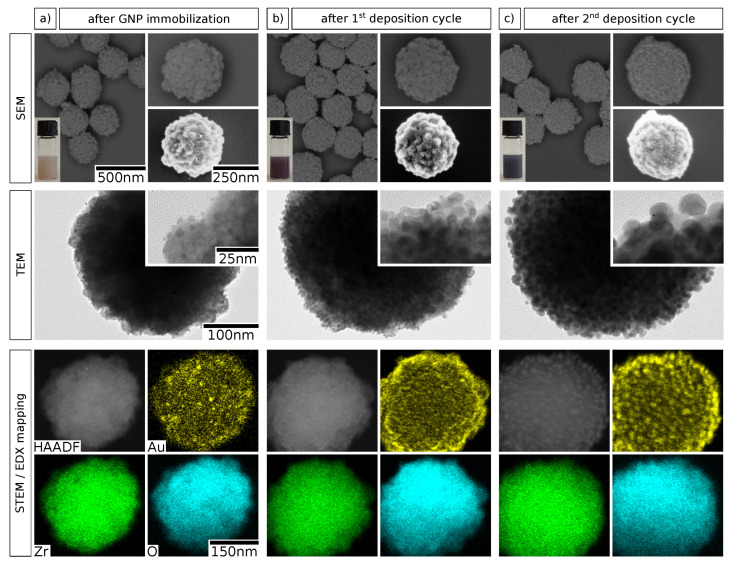
Electron microscopy characterization of zirconia@gold composite particles after GNP immobilization and subsequent reductive deposition of gold. The micrographs show column-wise the particles (**a**) after GNP immobilization, (**b**) after the first gold deposition cycle, and (**c**) after the second gold deposition cycle. The fist row shows SEM images obtained with an ESB detector (left and top right) and an in-lens detector (bottom right), as well as photographs of the respective particle suspensions (bottom left). The second row shows TEM images at higher magnifications. In the third row, elemental maps of gold, zirconium, and oxygen, obtained by spatially resolved EDX, are shown along with the respective HAADF-STEM micrographs. Different individual particles from the same sample are shown in each column. The scale bars shown in the left column apply to the corresponding images in all three columns.

In a consecutive set of experiments, we studied the deposition of additional gold onto the zirconia particle surface to enable the growth of a granular gold shell. Preliminary attempts to deposit gold onto bare zirconia particles by seeded reduction generally resulted in uncontrollable nucleation of gold and the formation of large aggregates in solution. Therefore, we propose that the precedent immobilization of GNPs on the particle surface as nucleation seeds is an indispensable step in this context. Accordingly, the deposition of additional gold onto GNP-decorated zirconia particles was achieved using a seeded growth method, inspired by previously published protocols for the deposition of platinum nanoshells [3,36]. A sample of GNP-decorated zirconia particles was obtained using the optimal parameters identified above (0.01 M and use of AMPA) and served as starting material. Here, elemental gold was deposited from aqueous solution of chloroauric acid and potassium carbonate (“K-gold”) by reduction with ascorbic acid, whereby the immobilized GNPs served as nucleation seeds. After purification, this cycle was repeated once to deposit additional gold onto the particle surface. The obtained samples were characterized using SEM, transmission electron microscopy (TEM), and energy-dispersive X-ray spectroscopy (EDX) in combination with high-angle annular dark field scanning transmission electron microscopy (HAADF-STEM). Representative electron micrographs, photographs, and elemental maps are depicted in Figure 4.

A comparison of the SEM images indicates a substantial and gradual growth of the immobilized GNPs with every deposition cycle. This finding is confirmed by the TEM images, where the increase in GNP size from ∼3 to ∼15 nm over two deposition cycles of seeded growth is evident. Additionally, the color of the particle suspension changes significantly from faint brownish over red/purple to dark violet/black, as shown by the insets. The colors arise from SPR excitation and the first change from brownish to red/purple indicates the growth of GNPs and decreasing interparticle distances [26]. Moreover, the dark violet/black color observed after two deposition cycles supposedly signifies starting GNP coalescence and marks the onset of the formation of a metal nanoshell [1,36]. The EDX mappings of the three deposition stages of GNP growth additionally visualize the controlled nature of the gold deposition process. Finally, the incremental gold deposition was evaluated by EDX quantification, as summarized in Table 1. The molar fraction of gold relative to those of zirconium and oxygen is very low after GNP immobilization. The value of (0.9±0.1) atom% translates to a weight fraction of (4.1±0.5) wt%, which is slightly higher than the gold content of (2.5±0.5) wt% observed for a similar sample in the first study (see Figure 2 and Table S1). However, the results are in reasonable agreement considering that the reproduced sample was obtained by a scale-up of the original synthesis and the fact that the values originate from very different characterization methods. As expected, the gold fraction is increased to 4.0 and 9.0 atom% after the first and the second deposition cycle, respectively. We suppose that more gold is deposited during the second cycle because the overall gold surface area is higher as a consequence of GNP growth during the first cycle. Thus, the number of potential nucleation sites for gold reduction is expected to be significantly higher. The data in Table 1 confirm the conclusions drawn from the findings based on the various imaging methods (see Figure 4) and demonstrate that the method allows for accurate control over the deposited amount of gold.

The chosen experimental conditions for seeded growth deposition enable the controlled seeded growth of immobilized GNPs on zirconia mesoparticles. Significant surface-attached or separate gold aggregates, which would indicate undesired secondary nucleation in solution, were not observed in any of the applied characterization methods. Although the reaction conditions have not yet been optimized systematically, the presented method and reaction parameters appear suitable for growing contiguous gold nanoshells onto zirconia mesoparticles.

## 3. Conclusions

The results presented in this work demonstrate effective immobilization of GNPs and well-controllable deposition of gold on spherical zirconia particles. We showed that seeded-growth known from the synthesis of gold nanoshells on silica spheres [1] can be adapted to the gold deposition on zirconia spheres provided that suitable means for efficient GNP immobilization are applied, i.e., a suitable adjustment of the pH and addition of the coupling agent AMPA. The systematic study with respect to the impacts of hydrochloric acid concentration and use of AMPA during GNP immobilization contribute to understanding the electrostatic and molecular interactions governing the coupling of GNPs to metal oxides. Furthermore, our novel synthetic approach provides a very useful tool for the preparation of metal ceramic composites that may be relevant to various fields of applications. Building up on the findings reported in this communication, we currently examine the suitability of the reported method for the deposition of thicker contiguous gold nanoshells as well as improvements concerning its complexity and efficiency. Moreover, the characteristic behavior in catalytic processes is being investigated in order to identify the general suitability as a catalyst material and potential drawbacks and advantages in comparison with other catalyst materials.

## 4. Materials and Methods

Chloroauric acid trihydrate (99.99%), L-ascorbic acid (99%), potassium carbonate (99.997%), 1-propanol (99.9%, anhydrous), and zirconium(IV) *n*-propoxide (70 wt% in *n*-propanol) were obtained from Alfa Aesar (Kandel, Germany). Aminomethylphosphonic acid (≥97%) was from TCI Chemicals (Tokyo, Japan), sodium hydroxide solution ( 0.1 M) and pH-indicator strips (MQuant 0–2.5, 2.5–4.5, 4.0–7.0) were from Merck (Darmstadt, Germany). 1-butanol (99.5%) and ethanol (99.5%, extra dry) were ordered from Acros Organics (Geel, Belgium). Icosanoic acid (≥99%), hydroxypropyl cellulose (99%, 20 mesh, $M_{w}$ = 80,000 g mol^−1^), and tetrakis(hydroxymethyl)phosphonium chloride (80% in water) were purchased from Sigma Aldrich (Munich, Germany). Ethanol (99.8%) and hydrochloric acid (37%) were ordered from VWR (Darmstadt, Germany). Ultrapure water (Elga LabWater, Purelab flex 2, 18.2 MΩcm, <5 ppb TOC) was used for all procedures.

The sol-gel synthesis of zirconia mesoparticles was published recently by our group [24]. Briefly, zirconium *n*-propoxide was added quickly to a vigorously stirred ethanolic solution containing icosanoic acid, hydroxypropyl cellulose, and water and the mixture was heated to 55 °C. After 3 h the opaque suspension was transferred into ice-cold 1-butanol and purified by repeated centrifugation, yielding spherical particles with a mean diameter of (384±36) nm. The obtained sample was subsequently calcined at 600 °C for 3 h and, after grinding, redispersed in water using an ultrasonic bath.

A GNP sol was prepared following the method reported by Duff et al. [25], by injection of 2 mL of a freshly prepared aqueous 25 mM chloroauric acid solution into a mixture of 45 mL water, 8.9 μL tetrakis(hydroxypropyl)phosphonium chloride solution (80 wt%), and 3 mL of a 0.1 M sodium hydroxide solution. After 30 min of extended stirring the brownish GNP sol was aged at 4 °C for 12 h in the dark and used without further purification. The obtained particles had a mean diameter of (2.8±0.9) nm, as determined by TEM.

The GNP immobilization procedure is illustrated in Figure 1. It was conducted equivalently for several samples with varying amounts of HCl and AMPA, so that the final HCl concentrations were 0, 10^−6^, 10^−5^, 10^−4^, 10^−3^, 0.01, 0.1 and 1 M. AMPA final concentrations were 6 mM for those samples where AMPA was employed. For each sample, 1 mL of the GNP sol was mixed with 100 μL of the readily redispersed suspension of calcined zirconia particles, followed by addition of AMPA, water, and HCl under constant stirring, according to Table S1 in the Supplementary Materials. After an extended stirring period of 30 min, the particles were purified by threefold centrifugation (10,000× *g*, 5 min), decanting of the supernatant, and redispersion in 1.5 mL water. The pH of each sample was determined by examining the first supernatant using pH-indicator strips.

Deposition of additional gold was done by a seeded growth method. GNP-decorated particles obtained by immobilization at a HCl concentration of 0.01 M and an AMPA concentration of 6 mM were reproduced in larger volume and used here as starting material. First, an aqueous solution of 0.9 mM potassium carbonate and 0.2 mM chloroauric acid (commonly referred to as “K-gold” [37]) was prepared, stirred for 30 min, and aged at 4 °C for 24 h in the dark. Per deposition cycle, 5 mL of the now colorless solution were added dropwise to a mixture of 2 mL dispersion of purified GNP-decorated zirconia particles, 8 mL water, and 50 μL of a freshly prepared 0.1 M L-ascorbic acid solution, at a constant rate of 0.3 mL h^−1^ under vigorous stirring. After complete addition, stirring was continued for 1 h. Subsequently, the samples were purified by repeated (3×) centrifugation (10,000× *g*, 10 min), decanting of the supernatant, and redispersion in 2 mL water.

SEM characterization was performed using a GEMINI LEO 1550 HRSEM from Carl Zeiss (Oberkochen, Germany), operating at an acceleration voltage of 2 kV and gas pressure of <10^−5^ mbar, equipped with an in-lens and an ESB detector, and using polished Si wafer substrates. TEM, HAADF-STEM and EDX mapping were conducted using a JEM 2200 FS from JEOL (Tokyo, Japan) and carbon-coated copper grids as specimen substrates. For elemental mapping and quantification, the K${}_{\alpha 1}$ signals of zirconium and oxygen and the L${}_{\alpha 1}$ signal of gold were evaluated. AAS analysis was carried out using a ContrAA 700 from Analytik Jena (Jena, Germany). Sample preparation was done by drying 250 μL of a sample, weighing, and fusion of the dry material by boiling in 4.0 mL aqua regia (caution: hazardous reagent!) for 1 h. For the measurements, 5.0 mL of a palladium magnesium nitrate (0.1%/0.05%) solution were used as a matrix modifier. The signal at a wavelength of 242.7 nm was evaluated for quantification. UV/vis/NIR spectroscopy was conducted using a Cary 5000 from Agilent (Santa Clara, CA, USA) with an integrated sphere accessory and a center cuvette mount. Samples were prepared by dilution of the obtained particle suspensions by the factor 2 and transfer into standard quartz cuvettes. Water was used for spectral background subtraction.

## Figures and Tables

**Table 1:** EDX elemental analysis of zirconia@gold composite particles at different stages of gold deposition. The values are given in atom% relative to the sum of the three quantified elements, i.e., oxygen, zirconium, and gold. The data were obtained by evaluation of single particles or groups of a few particles in close proximity. Given uncertainties represent the standard deviations of multiple measurements.

Element	After GNP Immobilization	After 1st Deposition Cycle	After 2nd Deposition Cycle
O	65.1 ± 0.0	49.2 ± 6.4	54.1 ± 2.5
Zr	34.0 ± 0.1	36.8 ± 0.8	36.9 ± 1.8
Au	0.9 ± 0.1	4.0 ± 0.1	9.0 ± 0.7

## Supplementary Materials

The following are available online at https://www.mdpi.com/2079-4991/10/6/1197/s1, Table S1: Summary of synthesis conditions and characterization results for all GNP immobilization experiments, Figure S1: SEM and photographic images for all samples of GNP immobilization study, Figure S2: UV/vis/NIR spectra and quantification results for all samples of GNP immobilization study.

## Abbreviations

The following abbreviations are used in this manuscript:

AAS	atomic absorption spectroscopy
AMPA	aminomethylphosphonic acid
ESB	energy selective backscatter
GNP	gold nanoparticle
HAADF	high-angle annular dark-field
SEM	scanning electron microscopy
STEM	scanning transmission electron microscopy
SPR	surface plasmon resonance
TEM	transmission electron microscopy
TOC	total organic carbon
w/o	without
