# Supplementary material for: Seeded Growth Synthesis of Zirconia@Gold Particles in Aqueous Solution
Source: Nanomaterials (Basel). 2020 Jun 19;10(6):1197. doi: 10.3390/nano10061197 (PMC7353092; doi:10.3390/nano10061197)
Supplement: Supplementary file 1 [file nanomaterials-10-01197-s001.pdf]

Supplementary Materials: Seeded Growth Synthesis of Zirconia@Gold Particles in Aqueous Solution

Gregor Thomas Dahl [1], Jan-Dominik Krueger [1], Sebastian Döring [1,†], Horst Weller [1,2] and Tobias Vossmeyer [1,*]

Table S1. Summary of synthesis conditions and characterization results for all GNP immobilization experiments. The concentrations of HCl, AMPA, and the particle concentration after purification are denoted by c_{HCl}, c_{AMPA}, and c_{p}, respectively. w_{Au} is the gold weight fraction determined by AAS, A_{450} represents the absorbance at 450 nm in UV/vis/NIR spectroscopy. Uncertainties are determined by the weighing error for c_p and by the weighing error or the standard deviation of double measurements for AAS (whichever is larger). All pH values were obtained with a tolerance of ±0.15.

synthesis				characterization		
**sample**	c_{HCl} / M	c_{AMPA} / mM	**pH**	c_{p} / g L^{-1}	w_{Au} / wt%	A_{450}
0	1	-	0.0	417 ± 33	9.83 ± 0.78	0.618
0A	1	6	0.0	550 ± 24	13.10 ± 0.54	0.989
1	0.1	-	1.0	433 ± 33	3.53 ± 0.09	0.343
1A	0.1	6	1.0	467 ± 47	5.22 ± 0.43	0.448
2	0.01	-	2.5	533 ± 47	0.98 ± 0.07	0.194
2A	0.01	6	2.2	450 ± 71	2.52 ± 0.45	0.282
3	10^{-3}	-	3.6	833 ± 33	0.37 ± 0.00	0.100
3A	10^{-3}	6	3.3	833 ± 33	0.81 ± 0.01	0.189
4	10^{-4}	-	4.4	800 ± 141	0.07 ± 0.01	0.055
4A	10^{-4}	6	3.6	917 ± 118	0.58 ± 0.08	0.148
5	10^{-5}	-	5.5	850 ± 118	0.07 ± 0.01	0.046
5A	10^{-5}	6	4.2	1067 ± 47	0.49 ± 0.02	0.133
6	10^{-6}	-	6.1	850 ± 71	0.09 ± 0.00	0.041
6A	10^{-6}	6	4.7	583 ± 33	0.74 ± 0.02	0.146
7	-	-	6.5	500 ± 33	0.11 ± 0.00	0.045
7A	-	6	4.7	650 ± 71	0.79 ± 0.09	0.158

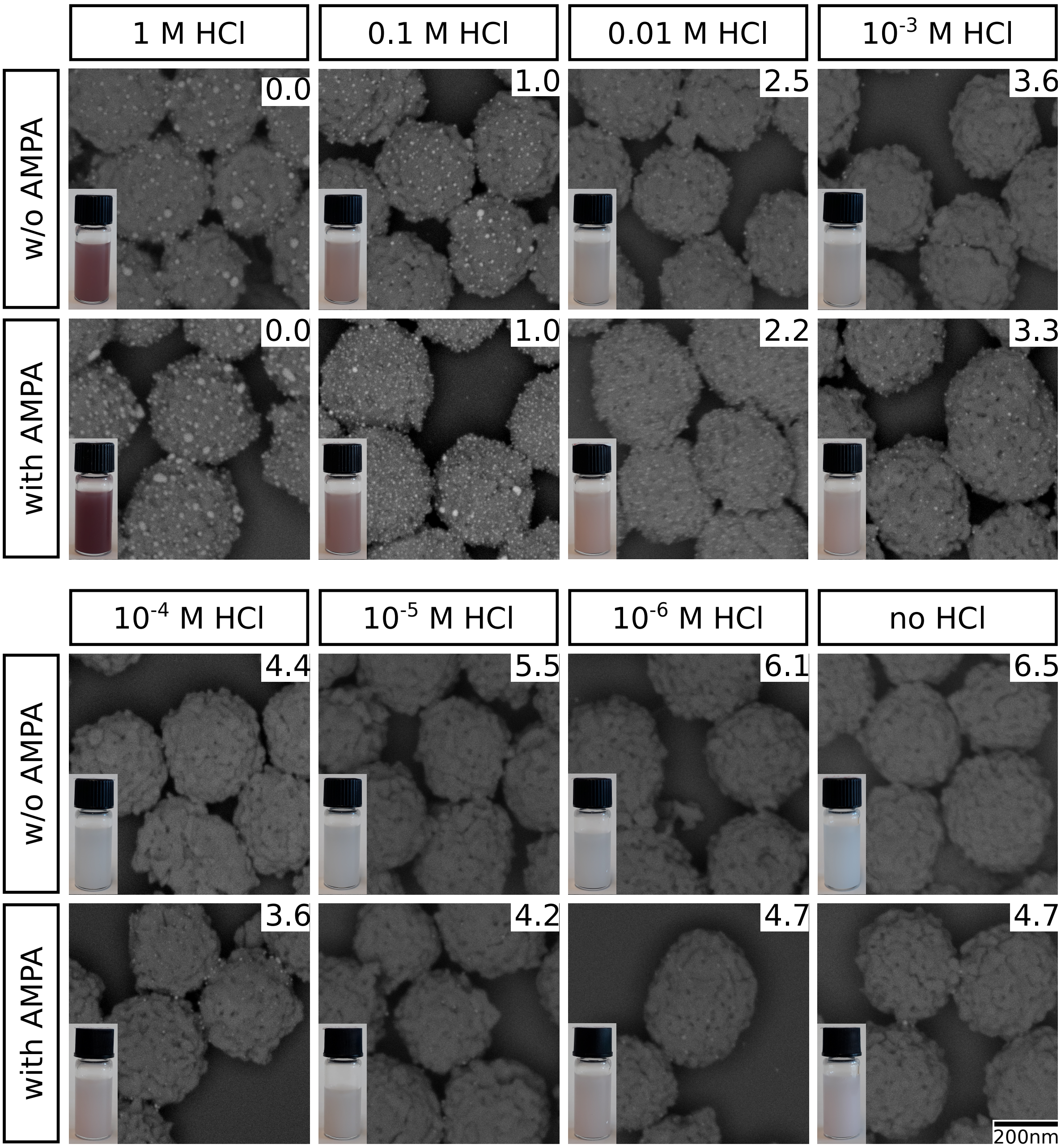

Figure S1. SEM and photographic images for all samples of GNP immobilization study. The samples correspond to those specified in Table S1, differing in HCl and AMPA concentrations during GNP immobilization. SEM images were obtained using an ESB detector, yielding a strong contrast between zirconia (dark) and gold (bright). Corresponding pH values are reproduced in the top right corners for convenience.

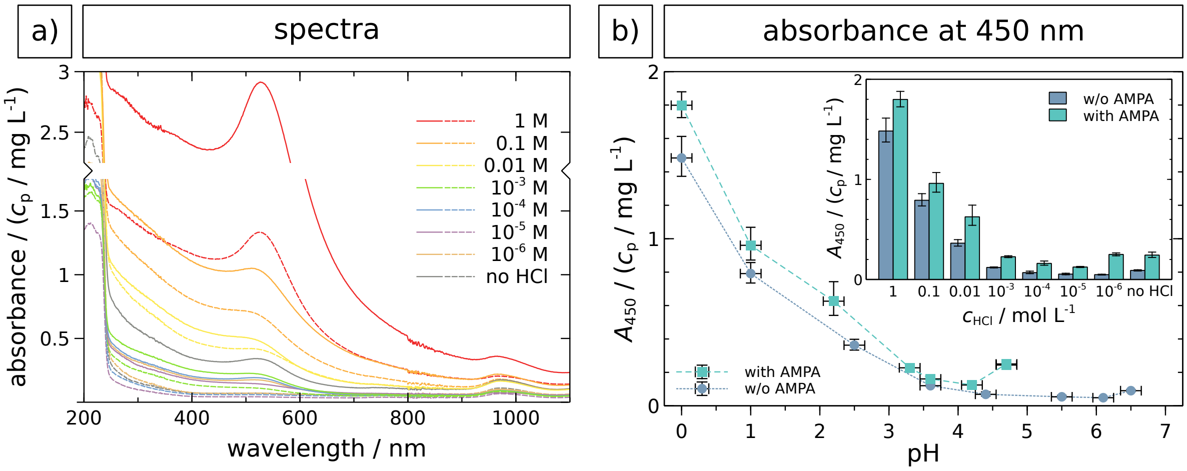

Figure S2. UV/vis/NIR spectra and quantification results for all samples of GNP immobilization study. The data were corrected for deviations in particle concentrations after purification c_p, as indicated in Table S1. **(a)** Corrected absorbance spectra for samples obtained in presence (solid lines) and absence (dashed lines) of AMPA. **(b)** Corrected absorbance at 450 nm as a function of pH (main plot) and HCl concentration (inset). Vertical error bars indicate the weighing error or standard deviation of double measurements, whichever is greater. Horizontal error bars represent the tolerance of pH measurements.
